# CMEO: a metadata-centric ontology for clinical studies exploration and harmonization assessment

**DOI:** 10.1186/s12911-025-03272-5

**Published:** 2025-12-06

**Authors:** Komal Gilani, Wei Wei, Christof Peters, Marlo Verket, Hans-Peter Brunner-La Rocca, Enrico Nicolis, Martina Colombo, Katharina Marx-Schütt, Visara Urovi, Michel Dumontier

**Affiliations:** 1 Institute of Data Science, Maastricht University, Maastricht, Netherlands; 2 Department of Acute Brain and Cardiovascular Injury, Institute for Pharmacological Research Mario Negri IRCCS, Milan, Italy; 3 Cardiovascular Research Institute Maastricht (CARIM), Maastricht University, Maastricht, Netherlands; 4 Department of Internal Medicine I, University Hospital RWTH Aachen, Aachen, Germany

**Keywords:** Ontology-driven data management, Metadata standardization, Privacy-preserving harmonization, Data interoperability

## Abstract

The integration of clinical research data across various institutions faces hurdles due to differing definitions, inconsistent terminologies, and inadequate support for interoperable metadata. While biomedical ontologies offer valuable tools for structuring clinical data, they have not yet been fully utilized for creating comprehensive metadata descriptors, such as variable semantics, statistical summaries, and governance elements essential for data discovery and alignment. We present the Clinical Metadata Exploration Ontology (CMEO) that builds upon well-established ontologies to provide a cohesive representation of study designs, data elements, exploratory statistics, and data reuse permissions. CMEO facilitates semantic querying for study exploration and comparison of data elements across studies, particularly when individual-level data cannot be shared. We demonstrate its utility using metadata from five studies: four heart-failure studies and one wearable-based type 1 diabetes study. After serializing, we executed SPARQL queries that operationalized study-level discovery, variable alignment across studies, and governance-constrained reuse. This FAIR-compliant, metadata-driven integration across heterogeneous sources enables scalable, privacy-conscious research and underpins federated clinical data exploration.

**Supplementary Information:**

The online version contains supplementary material available at 10.1186/s12911-025-03272-5.

## Introduction

Data integration has emerged as a major challenge across various scientific disciplines, driven by increasing volumes, variety, and complexity of data [1]. This challenge is particularly pronounced in clinical research, a cornerstone of biomedical science, where multi-center collaborations increasingly require the integration of heterogeneous datasets for secondary analysis, meta-analysis, and predictive modeling [2, 3]. These datasets often originate from diverse sources, such as electronic health records (EHRs), clinical trial systems, registries, and observational studies, with each source employing distinct data models, terminologies, and documentation practices.

To standardize such data, researchers typically utilize Extract–Transform–Load (ETL) pipelines to convert local data formats into common data models (CDMs), such as the Observational Medical Outcomes Partnership (OMOP) [4, 5] and the Health Level Seven (HL7) Fast Healthcare Interoperability Resources standard (FHIR) [6]. ETL processes are essential for the transformation and movement of individual patient data; however, these pipelines often require significant time for development, entail high maintenance costs, and present scalability challenges when study protocols and semantic conventions differ across institutions. Previous research on metadata-driven transformation underscores that ETL logic, when encoded manually, is prone to instability across varying contexts [7, 8].

Concurrently, access to individual-level patient data (IPD) is considerably constrained due to privacy regulations, ethical considerations, and institutional policies [9]. Researchers endeavoring to assess the relevance of studies, evaluate compatibility, or identify variables for harmonization frequently lack the opportunity to examine the raw data. Within such contexts, metadata—broadly defined as “data about data”—emerges as the primary means by which study information can be discovered, interpreted, and compared [7]. Comprehensive and well-structured metadata are indispensable not only for facilitating data integration and reuse, but also for enabling the scalable and privacy-preserving exploration of sensitive data [10].

This orientation aligns with FAIR Guiding Principles, which emphasize high-quality, machine-actionable metadata rather than unrestricted data dissemination [11]. According to FAIR principles, digital objects are required to be Findable, Accessible, Interoperable, and Reusable. Within restricted clinical environments, this necessitates a focus on metadata stewardship, which involves the utilization of standard terminologies, persistent identifiers, provenance, and well-defined access conditions. The integration of semantic structure facilitates the alignment and querying of datasets across various organizations and platforms, transforming isolated resources into interoperable assets for federated, multi-site secondary analyses.

In spite of the movement towards metadata-driven infrastructures, existing standards and models frequently prove inadequate in supporting the structured metadata essential for the exploration of clinical studies and the harmonization of data. For example, ISO/IEC 11,179 provides a general framework for metadata registries but lacks formal semantic relationships necessary for variable-level alignment across studies. The Clinical Data Interchange Standards Consortium (CDISC) [12] has proposed two major standards for clinical research: the Study Data Tabulation Model (SDTM) for structuring tabular study data [13], and the Operational Data Model (ODM) [14] for representing metadata and patient-level information in an exchangeable format. While both are well-suited for clinical trials, they are less applicable to observational or registry-based research. OMOP CDM [4, 5] and FHIR [6], facilitate IPD harmonization but offer limited support for explicitly modeling metadata relationships or enabling ontology-driven integration across heterogeneous sources [4, 5, 15]. In practice, research teams often rely on ad hoc local Knowledge Organization Systems (KOSs) for annotating datasets. These systems consequently lack interoperability and hinder data reuse across institutions.

To address this gap, ontology-based approaches have emerged as powerful tools for formalizing domain knowledge and promoting semantic interoperability. Biomedical ontologies such as the Ontology for Biomedical Investigations (OBI) [16], the Statistical Methods Ontology (STATO) [17], Relation Ontology (RO) [18], Information Artifact Ontology (IAO) [19], and the Ontology for Biomedical and Clinical Statistics (OBCS) [20] provide reusable structures for modeling study protocol, analyses, and outcomes. Similarly, the Data Use Ontology (DUO) [21] defines terms for expressing data access permissions and reuse constraints.

To the best of our knowledge, existing ontologies provide complementary yet partial coverage: some model data elements, some represent statistical methods and results, and some capture study provenance. No single ontology integrates these layers into a unified framework that directly supports exploratory data analysis (EDA) and cross-study compatibility, necessitating additional alignment and extensions [7].

We address the lack of metadata-centric frameworks for clinical study exploration and cross-studies variable comparability by introducing the Clinical Metadata Exploration Ontology (CMEO). It systematically reuses and extends existing ontologies such as OBI, STATO, OBCS, and DUO to provide formal representations of study design, variable-level descriptors, exploratory data summaries, and governance metadata. This semantic model enables metadata-driven querying of study characteristics and the alignment of semantically and statistically related variables across cohorts without requiring access to IPD.

In this study, we consider ETL as the subsequent execution phase and implement a metadata-first approach characterized by a formal and machine-actionable layer that preemptively encapsulates structural, semantic, and administrative representation. We demonstrate that the framework, centered on metadata, enables the discovery and comparability of studies in environments with restricted access and facilitates ETL by generating consistent mapping and transformation specifications when access is permitted. Overall, the main research question of this study is:

*Can a FAIR-aligned metadata-driven ontology model enable study exploration and cross-study variable alignment without access to individual data?*

CMEO has been developed and deployed within the context of the iCARE4CVD (Improved Cardiovascular Risk Stratification through Personalized Remote and Digital Interventions) initiative [22, 23], which aims to standardize and harmonize metadata across more than 50 cardiovascular studies. This deployment demonstrates CMEOs scalability and relevance for large-scale, real-world research environments. Furthermore, CMEO is being integrated into a semantic cohort exploration platform^1^, enabling users to query, manage, and access standardized metadata across heterogeneous clinical datasets.

The remainder of this paper is organized as follows. Section “Methods” describes the ontology development methodology and case study design. Section “Results” presents the results. Section “Discussion” discusses key findings, and limitations. Related work is presented in Sect. “Related work”. Finally, section “Conclusion” concludes the paper and outlines directions for future work.

## Methods

The CMEO ontology is designed to support metadata-driven study exploration and alignment feasibility (metadata-level harmonization) in privacy-preserving settings where IPD access is restricted. It builds on established biomedical ontologies such as OBI, STATO, and OBCS to provide a semantic representation of study protocol, variable-level descriptors, statistical summaries, and data use conditions. By aligning data elements with standardized terminologies such as Systematized Nomenclature of Medicine – Clinical Terms (SNOMED CT) [24], Anatomical Therapeutic Chemical (ATC) Classification (ATC) [25], International Classification of Diseases (ICD), 10th Revision [26], RxNorm [27], Logical Observation Identifiers Names and Codes (LOINC) [28], OMOP CDM [4, 5], and CDISC [12, 29], CMEO enhances metadata interoperability and enables scalable data harmonization assessment across heterogeneous datasets. We adopted an iterative, NeOn ontology engineering approach [30], which consists of the following phases: (1) knowledge acquisition; (2) ontology requirements specification; (3) ontology modularization; (4) ontology formalization; and (5) ontology verification. In this work, we present ontology verification results in section “Results”, where we demonstrate how CMEO satisfies its intended use cases through SPARQL Protocol and RDF Query Language (SPARQL)-based querying over metadata from five studies.

### Knowledge acquisition

The first phase focused on identifying metadata representation needs within the iCARE4CVD initiative. iCARE4CVD is a European public-private partnership aimed at improving the prevention and treatment of cardiovascular diseases through personalized, data-driven approach. The project brings together over 30 partners across academia, industry, and healthcare systems to enable the reuse and integration of real-world data from more than 50 multi-national observational and interventional studies. Its overarching goal is to support precision medicine and predictive modeling by harmonizing heterogeneous data sources across different populations, study designs, and health systems.

In this stage, we focused on gathering, organizing, and exploring metadata from multiple studies and understanding existing biomedical data standards, terminologies, and ontological models relevant to clinical research and healthcare. One of the key challenges in iCARE4CVD is that data integration across studies is hindered by differences in study design, variable naming, coding schemes, measurement units, and visit information. While the project aims to enable remote analyses on IPD, such semantic heterogeneity complicates exploration and alignment across studies. One approach to address this challenge is to develop harmonized metadata representations that support study discovery and variables alignment.

#### Data collection and exploration

**Fig. 1:**
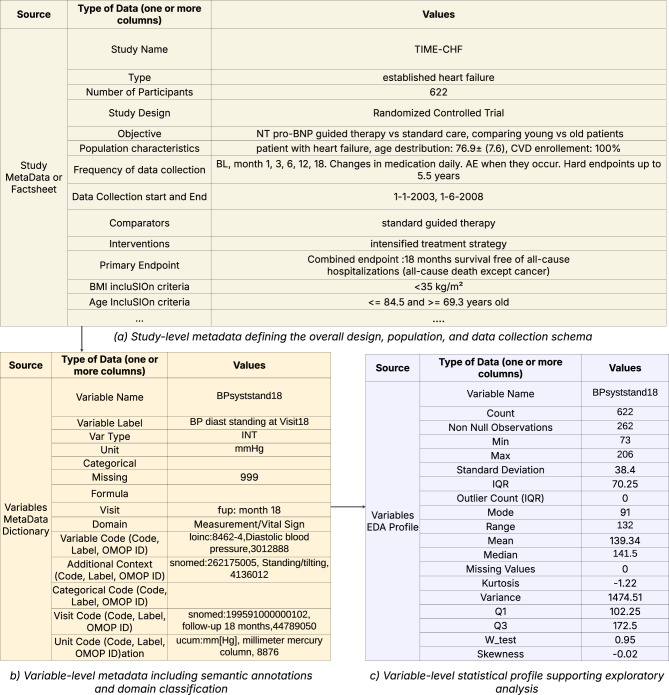
Sample metadata collected from clinical studies

As part of the iCARE4CVD initiative, we systematically curated and analyzed metadata from a diverse set of clinical studies, emphasizing core attributes including study design and protocol, selection criteria, wearable devices, data element (variable) descriptions, allowed categorical values, units of measurement, and time points at which data were collected. An overview of the curated metadata structure is provided in Fig. 1. Building on our prior work on metadata-driven vocabulary annotation [31], we mapped study-specific data elements to appropriate controlled terminologies, with domain experts and data custodians overseeing the validation process. The mapped data elements encompassed a wide range of domains, including medications, measurements, sign/symptoms, medical history, family history, procedures, medical devices, and visits information. The mapping strategy adhered to a domain-aware methodology informed by best practices in the OMOP CDM; for example, medications were linked to ATC or RxNorm, diagnoses to SNOMED CT, and laboratory tests to LOINC. By reusing terms from established vocabularies, we ensured that our mappings could be easily understood, reused, and integrated with other systems and datasets.

#### Exploration of existing ontologies

To inform the design of CMEO, we conducted a targeted ontology landscape analysis to identify existing ontologies capable of modeling critical aspects of clinical study metadata, including study protocol, data collection workflows, execution processes, and permission specifications. Following the NeOn methodology, we queried the ontology lookup service (OLS) [32] and NCBO BioPortal [33] using terms such as “study design”, “protocol”, “selection criterion”, “data element”, and “permission specification”. This analysis surfaced several widely used ontologies, including the OBI, STATO,OBCS, Semanticscience Integrated Ontology (SIO) [34], and DUO. As discussed in section “Related work”, each ontology provides partial coverage e.g., study modeling in OBI, statistical descriptors in STATO and OBCS, and governance modeling in DUO. However, none offers an integrated framework for representing study design, variable-level metadata, and data-access policies. These findings informed the scope and design decisions underpinning CMEO.

### Ontology requirements specification

The core design requirements were derived through iterative discussions with data analysts and domain experts from the iCARE4CVD consortium. The ontology must (i) enable detailed modeling of study-level and variable-level metadata to support harmonization and reuse, (ii) align data elements with biomedical terminologies (e.g., SNOMED CT, LOINC), (iii) enable structured querying over study protocol, statistical summaries, and governance metadata in compliance with FAIR principles, and iv) adhere to minimal ontological commitment, emphasizing reuse of existing terms, and introduce additional classes only when existing ontologies lack representational coverage.

***Scope and intended users***: The primary users of CMEO are clinical data analysts seeking to identify, describe, and compare studies based on metadata characteristics. The ontology, using only metadata, supports detailed exploration of study designs, variable specifications, and EDA without requiring access to IPD.

***Competency questions***: To validate the ontology’s design and ensure alignment with user expectations, we elicited a set of competency questions (CQs). These CQs reflect typical exploratory and harmonization related queries the ontology should support:

1. What are the protocol specifications of the randomized controlled trials?
2. Which studies investigated heart failure condition?
3. What are the population characteristics in different studies?
4. Which studies satisfy a given PICO (Population, Intervention, Comparator, Outcome) criteria ?
5. How are missing values encoded in study A?
6. Which categorical variables does Study A include, and what are their distributions?
7. Which variables are semantically similar between selected studies?
8. Which studies have variables measured with wearable devices?
9. What studies permit data access for research on congestive heart failure, subject to ethics-approval?

### Ontology modularization

In our ontology design, we followed two state-of-the-art recommendations. First, we adopted a top-level ontology, following Gangemi et al. [35], to achieve a conceptually rigorous, cognitively transparent, and efficiently exploitable knowledge organization system. Second, we adopt a three-layer ontology construction approach that distinguishes between upper (top), core, and domain/application ontologies to enhance semantic interoperability across diverse contexts,as proposed by Patel et al. [36]. This layered methodology is supported by the framework outlined in [37], which distinguishes between upper ontologies (for abstract grounding), domain ontologies (for disciplinary knowledge), and application ontologies (for task-specific instantiations). The concept of a hyperontology, as described by Kutz et al. [38], further reinforces this structure by promoting systematic reuse and semantic integration across layers of abstraction, particularly in biomedical domains.

To ensure conceptual rigor and semantic interoperability, CMEO adopts a modular architecture grounded in the Basic Formal Ontology (BFO) [39] as its upper-level framework. This decision follows the established modeling conventions of ontologies integrated into CMEO, including OBI, STATO, DUO, and IAO—all of which are BFO-compliant. BFOs clear ontological distinctions between continuants (entities persisting through time) and occurrents (entities unfolding in time) offer a principled foundation for modeling both static descriptors (e.g., data elements, units, permissible values) and dynamic processes (e.g., study execution, data collection).

Building upon this foundation, CMEO is designed using a three-tiered modular approach:

- Upper Layer: Inherits BFOs top-level categories to ensure high-level semantic alignment and consistency.
- Domain Layer: Reuses and minimally extends domain-relevant classes and relations from established biomedical ontologies—primarily OBI (processes, plan specifications, devices/specimens), OBCS (data collection), STATO (statistical descriptors), DUO (data use permission), together with IAO (information content entities) and RO ontology to model relationship between entities.
- Application Layer: Defines study-specific metadata instances in CMEO (e.g., data element, categorical values, temporal information, etc.) while linking them to biomedical terminologies via data standardization process.

This layered architecture enables CMEO ontology to remain lightweight, interoperable, and extensible, facilitating integration with existing knowledge frameworks while supporting flexible metadata representation across diverse study types.

### Ontology formalization

**Fig. 2:**
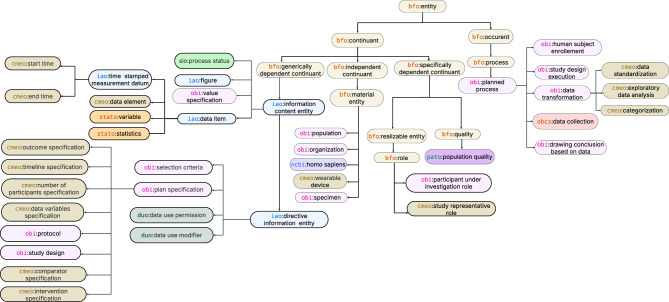
Core classes in CMEO ontology representing the clinical study life cycle from design to secondary reuse

We used Protégé 5.6.5 [40], an open source tool, for formalization and reasoning over ontology. Internal validity and consistency was checked using the semantic reasoner HermiT 1.4.3 [41]. The CMEO ontology formalizes core concepts essential for representing study designs, execution processes, data collection, and subsequent metadata exploration and reuse. The core classes, illustrated in Fig. 2, provide a structured representation of the clinical study life cycle, from protocol specification and execution to data collection, analysis, and secondary reuse.

*Study design*, as defined in OBI ontology, captures the prescriptive *plan specification* of a clinical investigation, including the *protocol, eligibility criteria, outcomes, study objectives, timeline specification, study variables specification*, and the planned *number of participants*. CMEO extends OBI with explicit representations of the study timeline, recorded-variable specifications, primary and secondary outcomes, and additional inclusion/exclusion criteria. Together, these elements provide the blueprint that guides the conduct of the investigation.

*Study design execution* is modeled as a *planned process* that realizes the *study design* and includes sub-processes such as *human subject enrollment* and *data collection*. The notions of input and output are employed strictly in the processual sense defined by OBI, and are represented by the object properties obi:has specified input and obi:has specified output, which relate a process to the entities it requires or generates, respectively. The *process* status and its subclasses are imported from SIO ontology under *information content entity* and linked to the execution process via iao:is about). CMEO extends the *start time* and *end time* as subclasses of *time stamped measurement datum*, each linked to the relevant process via iao:has time stamp.

*Material entity*, within BFO, denotes entities composed of matter, represented by the classes *object*, *fiat object part*, and *object aggregate*. Several subclasses of *material entity* are imported from OBI—including *organism*, *population*, *device*, *specimen*, and *organization*—and are extended in CMEO where needed. *Population* denotes groups of *Homo sapiens* serving as study participants, whereas *organization* denotes entities whose members enact roles in study planning and execution (e.g., *contact point*, *investigator*). *Device* and *specimens* participate in *data collection* process that yield *time stamped measurement datum*. The *Device* class is extended with subclasses *wearable device* and *sensor*. These subclasses may be linked to a participant via RO relation (ro:located in).

*Data element*, a new class introduced in CMEO, is modeled as a subclass of the IAO *data item* class and represents the specification of a study variable. Each data element has other information entities associated such as *categorical value specifications*, *measurement unit label*, and *visit measurement datum*.

*Data standardization* is modeled as subclass of OBI *planned process* class that obi:has specified input a *data element* and obi:has specified output one or more *codes* in biomedical terminologies. Separately, *categorization* is also modeled as a *planned process* that obi:has specified input a data element and obi:has specified output a *category* instance. CMEO further defines eight subclasses of *category* including: (1) *clinical event*; (2) *medication and therapies*; (3) *sign or symptom*; (4) *measurement* (including anthropometric, cardiovascular, blood-based, functional, and behavioral assessments, as well as clinical stages/scales); (5) *medical history and family history*; (6) *procedure*; (7) *visit*; and (8) *observation* (including demographics, compliance/adherence, lifestyle/behavior, and risk factors).

*Measurement datum*, as defined in IAO and OBCS ontology, refers to an individual observation and is a member of the *dataset* class corresponding to a specific *variable*. In the CMEO ontology, a *variable* is understood as the statistical representation of a *data element*, while a *measurement datum* is a single measured instance of that *variable*. This contrasts with the *measurement datum* modeling in OBCS, which considers each individual observation as a distinct *variable*. For example, when measuring “blood pressure” in 600 patients, “blood pressure” is treated as one *variable* comprising 600 *measurement datum*, with each patient’s measurement representing an individual observation of the same *variable* in CMEO. This allows for the analysis of distribution, central tendency, and variability in metadata across the population.

*Exploratory data analysis* is modeled as a subclass of the OBI *data transformation* class. It links to a *dataset* via obi:has specified input and yields a *statistic* via obi:has specified output. The resulting *statistic* is linked to the corresponding *variable* via iao:is about. It includes statistical attributes for both *categorical and continuous variables* such as *count, frequency distribution, skewness, and measures of central tendency*, which provide researchers with detailed insights into the distribution and characteristics of each *variable*.

*Data use permission*, imported from the DUO ontology, defines permissions for data reuse. It includes granular data-access policy classes, with subclasses such as *general research use*, *disease specific research*, *no restriction*, and *population origins/ancestry research only*. Additionally *data use modifiers* class represents additional conditions such as *ethics approval required, clinical care use, genetic studies only, geographical restriction* etc. These granular permission and use specifications ensures responsible and privacy-preserving secondary data use.

We primarily reuse object from RO, IAO, OBI, and DUO, and define exactly one data property for literals. Selected properties used include:

- *IAO*: iao:is about , iao:denotes, iao:has time stamp, iao:has measurement unit label etc.
- *RO*: ro:exists at, ro:concretize, ro:located in, ro:has participant, ro:has role, ro:has part etc.
- *OBI*: obi:has specified input, obi:has specified output, obi:has value specification
- *DUO*: duo:is restricted to
- *CMEO*: cmeo:has value (to represent literal values).

In addition to utilizing predicates from established ontologies, the ontology introduces several axioms to enforce explicit constraints and enhance the semantic precision of imported classes. For example, one axiom ensures that every dataset is explicitly linked to the study protocol. Formally, each *dataset* is an output generated by *data collection* process, which in turn concretizes a study *protocol*. This formal anchoring guarantees traceability and semantic rigor, ensuring each *datum* is justified by a clearly defined *protocol*. The axiom capturing this provenance constraint is stated as follows:

dataset equivalentTo: dataitem and (has member some (measurement datum and (is specified output of some (data collection and (concretize some protocol)))))

Another example of an added axiom addresses data format requirement for longitudinal data analysis. Formally, a *longitudinal data analysis* must take a *temporal dataset* as obi:has specified input. A *temporal dataset* is defined as a dataset containing *measurement datum* produced by *data collection* process that concretize study *protocol* explicitly incorporating *timeline specification*. This temporal constraint is formally represented by the following axiom:

temporal dataset equivalentTo: dataset and (has member some (measurement datum and (is specified output of some data collection and (concretize some (protocol and (has part some timeline specification))))))

### Case study design

To evaluate metadata representation for dataset exploration, we used metadata from five studies: four chronic heart-failure studies-two randomized clinical trials, one observational cohort, and one patient registry—and one diabetes type 1 wearable study. The trials include the Trial of Intensified versus Standard Medical Therapy in Elderly Patients with Congestive Heart Failure (TIME-CHF) [42] and the GISSI-HF study [43]—a randomized, double-blind, placebo-controlled trial investigating the effects of *n*-3 polyunsaturated fatty acids and the use of rosuvastatin in patients with chronic heart failure. The observational cohort, Aachen-HF [44], has been conducted at RWTH University Hospital Aachen and focuses on patients with established heart failure. The CHECK-HF registry is a multi-center initiative aimed at optimizing care according to the European Society of Cardiology (ESC) guidelines for chronic heart failure [45]. Additionally, we used the open-source D1NAMO dataset, which was acquired on 20 healthy subjects and 9 patients with type 1 diabetes [46], for wearable data representation [46]. The D1NAMO dataset is a publicly available multimodal resource designed to detect glycaemic events using non-invasive ECG pattern analysis.

As illustrated in Fig. 1, each data element and its associated attributes such as description, temporal information, measurement units, and permissible categorical values, were linked to corresponding standardized codes. In addition, statistical summaries for each dataset were generated by the data custodians using Python 3 scripts executed within a privacy-preserving environment. The metadata from five studies were modeled using CMEO, with Resource Description Framework (RDF) representations serialized in Turtle syntax to support semantic interoperability. Source tables (CSV) containing both study protocol metadata and variable-level (data element) metadata were transformed to CMEO-aligned RDF/Turtle by a Python 3 script and uploaded as named graphs (one per study) to the local SPARQL endpoint. The python 3 script for generating graphs and resulting named graphs are available as supplementary material in the public code repository [47].

To perform query retrieval, the generated Turtle files were uploaded to a SPARQL-compatible triple store, and the competency questions (CQs) were translated into SPARQL queries. Overall, All queries operates entirely on available metadata and does not access IPD. The following paragraphs present design of each CQ ; all executable SPARQL queries for CQ1–CQ9 appear in supplementary material.

In CQ1, the query operates over the study-level metadata graph, targeting *obcs:randomized controlled trial* and associated elements via ro:has part relationship. Both study design and protocol are modeled as subclasses of *obi:plan specification* and are therefore capable of being related through ro:has part relationship.

A common requirement in clinical research is to identify studies that investigate specific disease or disorder. For example, researchers conducting meta-analyses, cohort comparison, or external validations may seek studies involving disease-specific research. In CQ2, to identify studies investigating “heart failure”, the query tests whether the term appears in either the *obi:objective specification* or *obi:health status inclusion criteria*, using a simple substring match across both fields. This baseline does not perform synonym expansion or semantic reasoning but serves as a practical first-pass filter. Using standard vocabularies then enables more precise and semantically rich retrieval.

CQ3 extracts cohort descriptors that are produced by the subject-enrollment workflow and asserted as qualities of the resulting *population*. For each study graph, the SPARQL query follows the protocol’s eligibility criteria to the enrollment output (*obi:population*) and then collects its characteristics via ro:has characteristic (e.g., morbidity, sex and age distribution etc.). To provide a compact per-study synopsis, labels of the discovered qualities are aggregated using GROUP_CONCAT function, with light normalization applied for display.

In CQ4, we evaluate a PICO-based retrieval over study metadata. The criteria were operationalized as follows: Population—explicit reference to NYHA class II within the health-status inclusion criteria and age 60–85 years; Intervention—intensified therapy; Comparator—standard guided therapy; Outcome—survival. The SPARQL query encodes these constraints on the study-level metadata graph and employs GROUP_CONCAT to aggregate matched values per study.

Studies may encode missingness using various conventions such as “na”, “999” or “unknown”. Identifying these encodings at the metadata level allows researchers to pre-process data appropriately. The CQ5 query targets the TIME-CHF named graph to enumerate data elements and report their corresponding missing-value counts. Each *cmeo:data element* can be linked to one or more *cmeo:missing value specification* instances via obi:has value specification; the values used to indicate missingness are recorded as literals via cmeo:has value.

CQ6 examines categorical variables in the TIME-CHF named graph. The query follows the CMEO provenance pattern whereby a *cmeo:exploratory data analysis* takes as input an *iao:dataset* about a variable—either a *cmeo:binary class variable* or a *cmeo:multi class variable*—denoted by a *cmeo:data element*, and yields a *stato:statistic* that includes an *obcs:frequency distribution*.

While the previous CQs demonstrated how proposed ontology supports structured study retrieval through metadata-driven queries, CQ7 addresses a fundamental challenge in translational research and meta-analysis which is the identification and alignment of semantically and statistically similar variables across heterogeneous clinical studies to enable meaningful data integration. In real-world research settings, the data collected within a single clinical study are often insufficient for robust analysis and/or for building generalizable predictive models [48]. As a result, researchers increasingly rely on pooled analyses and federated data environments that draw upon IPD from multiple studies.

To demonstrate CQ7 in practice, We examined two large heart-failure studies—TIME-CHF and GISSI-HF—that differ in design, geography, and data collection. For retrospective harmonization the prerequisite is to determine whether variables are comparable and can be semantically aligned. CMEO supports this by linking each standardized data to unit, permissible values, and visit annotations. Using TIME-CHF as the source, we compared a curated set of clinically relevant variables against GISSI-HF and, via SPARQL, retrieved pairs sharing a common code or exhibiting semantically related representations. We extended matching beyond exact code equality by using hierarchical relations within and across vocabularies. Specifically, we inferred broader/narrower alignments via ontology links (e.g., is a, part of, subsumes) in SNOMED CT, LOINC, RxNorm and ATC, and performed cross-vocabulary mapping (e.g., ATC-RxNorm, SNOMED-RxNorm, SNOMED-LOINC etc.) using mappings available through the Athena vocabulary system [49]. For example, RxNorm “furosemide” was aligned to ATC “furosemide and potassium-sparing agents; systemic” based on established class–ingredient relationships.

To identify candidate alignments, the core SPARQL query searches two named graphs for data elements that (i) share a standardized code , (ii) belong to the same semantic category (e.g., measurement, condition, medication etc.), and (iii) are temporally aligned (e.g., baseline, follow-up). For derived variables such as estimated Glomerular Filtration Rate(eGFR) or Body mass index(BMI), we performed a post-processing check to confirm that the requisite inputs (e.g., serum creatinine, weight, height) were present in the metadata via standardized codes. When all inputs were available, we labeled the target as a potential match and marked it “(derived)” to indicate computability.

CQ8 targets studies with variables derived from wearable devices, which are increasingly used in longitudinal research to capture physiological and behavioral signals (e.g., heart rate, activity, sleep, glucose) at high cadence [50]. In CMEO, a *cmeo:wearable device* is linked to a *cmeo:sensor* via ro:has part, participates in an *obcs:data collection* process, yields an *iao:dataset*, and connects to *cmeo:data element* instances representing the recorded variables. The SPARQL query traverses these relations to identify studies containing wearable data.

In privacy-sensitive research environments, it is increasingly important to identify datasets that are eligible for reuse under well-defined ethical and legal conditions [51]. CQ9 addresses discovery of studies permitting research on congestive heart failure contingent on prior ethics approval. Such permissions are governed by consent processes and institutional access protocols. In this query, the dataset carries the DUO permission *duo:disease specific research*, linked to *snomed:congestive heart failure*, with the additional constraint *duo:ethics approval required*. These annotations are issued by a documented *cmeo:data use permission assignment* process, which records provenance and the responsible *organization*.

#### Harmonization assessment

We use the term harmonization assessment to denote a metadata-only procedure that would estimate, prior to any transfer or transformation of IPD, how readily a source data element can be aligned to one or more target elements across studies. The output is a specification report for each source–target pair. It captures the following attributes:

- **Match type**: Primary evidence for alignment, classified as code match (identical standard code), hierarchy-proximate (adjacent in a controlled-vocabulary hierarchy, derived (via a well-defined derivation) or none.
- **Unit compatibility**: Semantic compatibility of measurement units such as compatible, convertible, or incompatible. Unit normalization follows established unit system where available.
- **Visit alignment**: Temporal comparability of measurement context
- **Permissible values**: Comparability via standard-code matches and category-set overlap.
- **Derivation feasibility**: Whether the target can be computed from available inputs
- **Transformation rule**: Description of concise operations required for alignment
- **Alignment category**: Final feasibility class—complete match (identical), complete match (compatible),partial match (proximate), partial match (tentative), or not applicable; assigned by simple rules over the attributes above (see Table  1).

**Table 1:** Classification of harmonization categories with corresponding definitions, permitted actions, and illustrative examples

Category	Short definition	Example
Complete match (Identical)	Same construct and context (specimen, visit/timepoint, method), same value domain and unit; codes fully equivalent.	“heart rate at baseline (mmHg)” in datasets
Complete match (Compatible)	Same construct and context; only trivial normalization needed (unit conversion with known factor or 1:1 categorical label normalization).	Creatinine umol/L vs. mg/dL; “M/F” vs. “Male/Female”
Partial match (Proximate)	Same construct but categorical value sets differ in number or granularity; or minor contextual drift (e.g., baseline vs. enrollment window).	Smoking status (never, current) vs. (never, former, current).
Partial match (Tentative)	Candidate match needing clarification, normalization, or policy decision; often cross-type (binary$\leftrightarrow$multi, continuous$\leftrightarrow$categorical, qualitative$\leftrightarrow$structured).	bundle branch block (BBB) (yes/no) vs. BBB type diagnosed (left/right/right+anterior hemiblock/posterior hemiblock)
Not applicable	No meaningful mapping (different construct/intent) or target variable absent.	“Dietary sodium advice given?” vs. “Serum sodium (mmol/L)”.

It serves as a human-interpretable justification of the proposed alignment to parameterize downstream transformation when access and governance permit. Harmonization assessment is not encoded as Web Ontology Language (OWL) axioms within CMEO. In this manuscript we report only the complete match (identical). We have outlined the structure of a prospective harmonization specification to indicate the information required by complementary tooling. Compatible, proximate, and tentative alignments are computed in a post-processing stage and are therefore not detailed here.

## Results

The resulting CMEO ontology comprises 329 core classes, 79 of which are newly defined (Table  2). To demonstrate its practical value, we apply CMEO in a case study that showcases unified metadata representation, discovery, and cross-study variable comparability. The evaluation is organized around the competency questions (CQs) introduced in section “Ontology requirements specification”, reflecting typical researcher queries in restricted-access settings. Because CMEO is designed for privacy-preserving use, these questions are answered solely from metadata. The following subsections report the results for each CQ; query formulations are described in the Methods and supplementary material.

**Table 2:** Total core ontology terms used in CMEO as of Sept 8, 2025

Provenance	Ontology	Classes	Object Properties	Data Properties
Imported	BFO	34	0	0
	OBI	75	6	0
	OBCS	51	0	0
	IAO	40	7	0
	STATO	17	0	0
	DUO	20	1	0
	SIO	5	0	0
	Phenotype And Trait Ontology (PATO) [52]	7	0	0
	NCBI Taxonomy [53]	1	0	0
	Relation Ontology (RO)	0	28	0
New	CMEO	79	0	1
**Totals**		**329**	**43**	**1**

### CQ1: what are the protocol specifications of the randomized controlled trials?

The CQ1 query retrieves protocol elements for studies whose design is a randomized controlled trial (RCT)—including TIME-CHF and GISSI-HF—returning objectives, eligibility criteria, participant counts, and primary outcomes (see Table  3).

**Table 3:** CQ1 results show the key protocol elements for TIME-CHF and GISSI-HF (abridged)

Study	Element	Value
TIME-CHF	primary outcome	18-month survival free of all-cause hospitalizations and all-cause death except cancer
	health status inclusion criterion	heart failure, clinical signs or symptoms of chronic heart failure ( $\ge$ NYHA class II dyspnea on current therapy).
	objective specification	Comparative study with an intensified treatment strategy guided by NT-BNP levels against standard symptom-guided therapy in patients with chronic heart failure aged 60–74 years and those 75 years or older
GISSI-HF	primary outcome	Time to death and stroke.
	health status inclusion criterion	Heart failure.
	objective specification	whether long-term administration of *n*-3 PUFA and rosuvastatin is more effective than the corresponding placebo in reducing all-cause mortality and all-cause mortality or hospitalizations for cardiovascular reasons

### CQ2: which studies investigated heart failure condition?

Applying CQ2 across the five study graphs returned four studies—TIME-CHF, GISSI-HF, Aachen-HF, and CHECK-HF—each explicitly referencing heart failure in their protocol metadata (objectives and/or inclusion criteria). The D1NAMO wearable dataset was not retrieved, consistent with its focus on glycaemic event detection rather than heart failure (see Table  4).

**Table 4:** CQ2 results show studies investigating heart failure

study_name	description
TIME-CHF	established chronic heart failure
GISSI-HF	established heart failure
CHECK-HF	established heart failure
Aachen-HF	established heart failure

### CQ3: what are the population characteristics in different studies?

The query returned population summaries for TIME-CHF and GISSI-HF(see Table  5). Both studies enrolled patients with heart failure. TIME-CHF reports a sex distribution of 59.3% male and 40.7% female, with mean age of $76.9 \pm 7.6$ years; GISSI-HF reports 78.3% male and 21.7% female, with mean age of $67 \pm 11$ years.

**Table 5:** CQ3 results show the population characteristics of four studies

study_name	characteristic_type	characteristic_value
TIME-CHF	morbidity,mixed sex, age distribution	heart failure; male = 59.3% and female = 40.7%; $76.9 \pm 7.6$
GISSI-HF	morbidity, mixed sex, age distribution	heart failure; male = 78.3%; female = 21.7%; $67 \pm 11$

### CQ4: which studies satisfy a given pico criteria ?

The query returned a single study, TIME-CHF, satisfying all four PICO components (Table  6). The population corresponds to chronic heart failure with NYHA Class II and an age window of 60–85 years. The intervention is an intensified treatment strategy, contrasted with a comparator of standard symptom-guided therapy. The outcomes include survival endpoints. These results show that a PICO-driven query can retrieve eligible studies from metadata alone.

**Table 6:** CQ4 results show a study (TIME-CHF) satisfying all specified pico components

study_name	population	interventions	comparators	outcomes
TIME-CHF	clinical signs or symptoms of chronic heart failure (dyspnea new york heart failure classification class two or more on current therapy)	intensified treatment strategy	standard guided therapy	18-month survival free of all-cause hospitalizations and all-cause death except cancer

### CQ5: how are missing values encoded in study a?

**Fig. 3:**
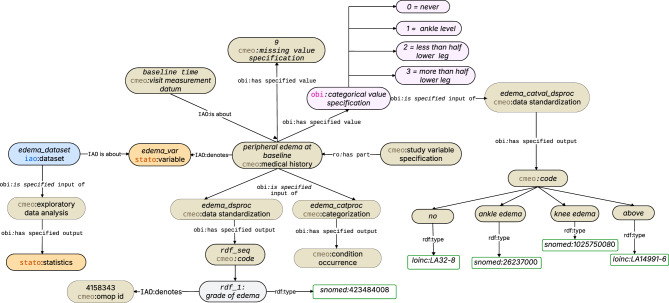
CMEO representation of the peripheral edema grade

The query enumerated missing-value encodings for TIME-CHF variables (Table  7). Some variables use a single value (e.g., edema uses 9; tdiarv_18 uses 0), whereas others use multiple (e.g., sodium uses 99 and 9999). These conventions inform downstream imputation, filtering, and alignment. Figure 3 illustrate the formal representation of missing value for variable “peripheral edema grade”.

**Table 7:** CQ5 results show missing-value encoding of three variables

variable	missing_value
edema	9
tdiarv_18	0
sodium	$99| 9999$

### CQ6: which categorical variables does study a include, and what are their distributions?

**Fig. 4:**
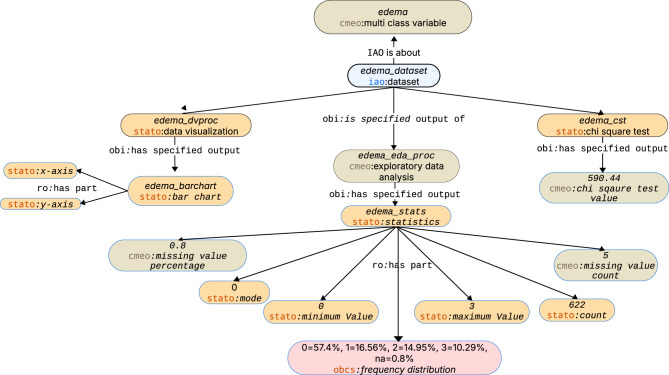
CMEO EDA representation of the peripheral edema grade

The query returned frequency distribution for three categorical variables in TIME-CHF dataset. The results includes rales1_01, sleeping_pill, and edema, each with observed class proportions (see Table  8). These metadata-level summaries support quick checks of class balance and identification of sparse or missing categories without accessing individual-level data. Figures 3 and 4 illustrate the standardization and the encoded distribution of the categorical variable “peripheral edema grade”, respectively.

**Table 8:** CQ6 results show the frequency distribution of three baseline variables

variable	fq_value
rales1_01	0 = 68.65%, 1 = 21.22%, na = 10.13%
sleeping_pill	0 = 59.49%, 1 = 40.51%
edema	0 = 57.4%, 1 = 16.56%, 2 = 14.95%, 3 = 10.29% na = 0.8%

### CQ7: which variables are similar between selected studies?

We identified 504 candidate variable pairs across medications, diagnoses, signs and symptoms, vital signs, laboratory measures, and endpoints (see Table  9). Two clinical experts independently reviewed and validated all matches. Matches arose through exact code concordance (e.g., hsCRP, NYHA_class, Gender), through hierarchical or class–ingredient relations (e.g., RxNorm furosemide aligned to the ATC class of furosemide and potassium-sparing agents; systemic), and through derived constructs when required inputs were present (e.g., BMI and eGFR, flagged as “(derived)”). Differences in representation were common. For instance, edema was recorded as a binary variable in one study and as a graded severity in the other. Such pairs were retained as harmonizable depending on the analysis plan.

**Table 9:** CQ7 results show similar variable pairs across TIME-CHF and GISSI-HF and their standardized representations

TIME-CHF	GISSI-HF	Standard Label
Dosebb	Dateno	Beta blocking agents| Atenolol
eGFR_cg_12	eGFR (derived)	Estimated creatinine clearance using cockcroft-gault formula
Mitrat	Mitrati	organic nitrates
Bundeltakblok_bb	Bb_latobb	Bundle branch block
Loop_dose	Dfurosem	Furosemide and potassium-sparing agents; systemic| Furosemide
hsCRP	hsCRP	C-reactive protein [mass/volume] in serum or plasma by high sensitivity method
hsTnT	hsTnt	Troponin t.cardiac panel [mass/volume] by high sensitivity method
Weight	PESOATT	Body weight
Frequentie	Fc	Heart rate
NYHA_class	NYHA	New york heart association classification
Angina	Preang	Angina pectoris
Edemahist	Edemperi	Grade of edema
Rales_01	Rantobas	Respiratory crackles
JVP	PVC	Distention of jugular vein| Central venous pressure
Hepatomegaly	Epamega	Large liver
BPdiast	Pad	Diastolic blood pressure
Fu_hf	Time_from_last_hosp	study endpoint
Gender	SESSO	Sex assigned at birth
RasDose	dlisino	Agents acting on the renin-angiotensin system| Lisinopril
BMI	BMI(derived)	Body mass index [ratio]
Age	age	Age
Diabetes	Prediab	Diabetes Mellitus

### CQ8: which studies have variables measured with wearable devices?

The query returned wearable-derived variables only for the D1NAMO dataset; none were identified in the four heart-failure studies. The retrieved variables included Breathing rate, Heart rate variability, Amplitude of breathing, ECG signal strength, and Skin temperature, all measured with the zephyr bioharness 3 (see Table  1^0^).

**Table 10:** CQ8 results show five observations from the D1NAMO study recorded using the zephyr bioharness 3 device

study_name	var_name	device_id
D1NAMO	Breathing rate	zephyr bioharness 3
D1NAMO	Heart rate variability	zephyr bioharness 3
D1NAMO	amplitude of breathing	zephyr bioharness 3
D1NAMO	ECG signal strength	zephyr bioharness 3
D1NAMO	Skin temperature	zephyr bioharness 3

### CQ9: what studies permit data access for research on congestive heart failure, subject to ethics-approval?

**Fig. 5:**
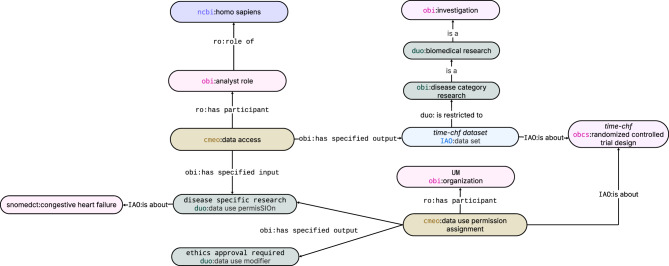
CMEO encoding of data use permissions in TIME-CHF study

As shown in Fig. 5, TIME-CHF permits research on congestive heart failure, conditional on prior ethics approval. This result derives from DUO annotations and linkage to the SNOMED CT concept “congestive heart failure.” CMEO can surface governance constraints during semantic discovery, while enforcement remains with external access-control systems.

## Discussion

While existing standards and data models have made significant progress in addressing aspects of structural or patient-level harmonization, they often fall short in representing the rich semantics required for studies alignment, governance-aware metadata exploration, and harmonization planning. Combining observational and clinical trial data enables a more comprehensive understanding of treatment effects and their generalizability. Clinical trials frequently exclude substantial segments of the population typically encountered in routine practice. Integrating both data sources allows researchers to assess the extent to which trial findings apply to real-world patient cohorts. This represents an important contribution of initiatives such as iCARE4CVD.

The CMEO is developed to bridge this gap by providing a comprehensive, ontology-based framework for modeling and querying clinical study metadata in a privacy-preserving manner. CMEO is aligned with the FAIR principles by ensuring that clinical study metadata are findable through persistent identifiers and semantic indexing, accessible under clear usage conditions in restricted settings, interoperable through reuse of established ontologies and standards, and reusable due to detailed semantic annotations that support exploration and comparison across studies. By modeling study protocol, data element semantics, exploratory data analyses, and data governance constraints, CMEO enables researchers to identify comparable cohorts, and assess metadata-based harmonization potential prior to initiating formal data access procedures. In federated research environments, where IPD access is restricted by institutional, legal, or ethical constraints, this capability becomes essential. Researchers can reason over structured metadata to assess study relevance and identify variable overlaps. When integrated with federated infrastructure such as the Personal Health Train [54], these metadata can inform decisions about whether, where, and how to execute analyses without moving IPD.

The adoption of a modular architecture was instrumental not only in ensuring semantic consistency with widely adopted biomedical ontologies but also in enabling CMEO to bridge conceptual gaps between protocol modeling, statistical representation, and governance. By combining static (information entities) and dynamic elements (processes) within a unified representational layer, the ontology advances the capacity of metadata ontologies to support reasoning over study design, temporal structure, and access permissions. These capabilities are rarely addressed in existing frameworks.

Competency questions CQ1 through CQ4 illustrate how the ontology facilitates fine-grained querying of protocol components, including eligibility criteria, study objectives, and outcome definitions. For instance, representing population qualities and temporal eligibility constraints enables more granular matching between study populations, advancing the precision of meta-analytic inclusion or external validation.

The modeling of data elements, including contextual attributes such as standard codes, units, and value sets, plays a pivotal role in assessing metadata-based variables alignment. The ontology captures both categorical and numerical variable distributions as part of EDA metadata (CQ5–CQ6), thus supporting preliminary assessments of cohort composition and statistical comparability. In CQ7, semantic richness allows identification of similar variables across two randomized controlled trials, demonstrating the practical value of semantic matching. CQ8 showed that only D1NAMO included wearable-derived variables, reflecting differences in study design and objectives rather than any limitation of representation.

In addition to structural and descriptive metadata, the ontology explicitly incorporates governance-level metadata through the integration of the Data Use Ontology (DUO). The ability to represent data use permissions and modifiers (CQ9), adds a practical layer of governance-aware metadata exploration. This is especially critical in distributed research infrastructure, where ethical compliance must be assessed prior to data access.

Although our evaluation focused on heart failure and diabetes studies, the modeling patterns introduced by CMEO are intended to be domain-agnostic. We expect CMEO to be applicable to additional clinical areas provided that comparable metadata are available—specifically: (i) study design and protocol specification, (ii) data metadata, and (iii) data-access policies. In domains with specialized assays or processes (e.g., imaging, genomics), minimal, modular extensions (additional subclasses or vocabulary mappings) may be required. This conditional generalizability reflects CMEOs role as a metadata-level framework rather than an IPD model. In routine use, CMEO is operationalized via a scripted workflow and does not require manual OWL editing. Effective deployment typically involves a data steward to curate metadata and a user with basic technical proficiency to execute the scripts. The principal burden is thus the quality of metadata curation, rather than tooling complexity.

Finally, the “harmonization assessment” denotes a metadata-level appraisal of cross-study alignment feasibility, distinct from the downstream execution of harmonization on IPD. CMEO faithfully represents what is provided—it is not an adjudicator of annotation correctness nor an inference engine for clinical equivalence—so heterogeneity in upstream annotations will directly condition the feasibility of alignment. By making such differences explicit, ontology can support principled judgment and the drafting of a prospective harmonization specification, while leaving operational transformations outside its scope. In practice, consistent reuse of controlled vocabularies improves annotation quality and increases the yield of alignable variables. Thus, CMEO provides a stable representational substrate on which complementary tools can operate, and closing the loop between annotation practices and assessment criteria is a key pathway to more effective, reusable harmonization planning.

### Limitations

Despite its contributions, the ontology is subject to certain limitations. An important consideration is the dependency on well-curated metadata. Studies with incomplete documentation or poorly defined variables may not benefit fully from this representational expressiveness. To address this, ongoing efforts are needed to encourage structured metadata authoring, supported by community guidelines and appropriate tooling for high-quality metadata creation. Moreover, careful planning is required during study design and data collection to ensure future usability in federated research settings that adhere to FAIR principles.

Additionally, although CMEO supports structured representation and retrieval of study variables via standardized codes, effective cross-study harmonization often requires an interpretive layer that links quantitative measurements to observable clinical signs. Biomedical terminologies are optimized for classification and documentation rather than for modeling such conditional equivalences grounded in clinical reasoning. For example, in our analysis the TIME-CHF variable “JVP” is annotated with the SNOMED term “Distention of jugular vein (o/e—jugular venous engorgement):, whereas the corresponding GISSI-HF variable “PVC” is mapped to “Central venous pressure”. Both index the same underlying physiology, “Right-atrial pressure”, yet they reside in different semantic branches and are not connected by existing hierarchical relations. In clinical practice, however, threshold-based interpretations are routine (e.g., jugular venous pressure $\geq$ 4 mmHg or central venous pressure $\geq$ 6 cm H$_2$O as evidence of mildly elevated right-atrial pressure). Because such correspondences depend on guideline context, assay/device characteristics, population, and unit conventions, we do not code them as OWL axioms in CMEO.

In practice, whether two variables can be treated as equivalent depends on context—study design, clinical intent, analytic objectives, and acceptable loss of granularity. For example, variables related only via a shared superclass (e.g., the drug class furosemide and potassium-sparing agents and the agent furosemide) may be harmonizable for class-based subgroup analyses yet inappropriate for dose-specific safety evaluations. Treating such links as global equivalences risks false positives where finer resolution is required. Harmonization logic should therefore remain context-aware and use-case-driven, with formalized rules serving as modular, transparent aids to expert-guided alignment rather than universal prescriptions.

Furthermore, CMEO is intentionally limited to metadata-level representations and does not encode individual-level clinical observations, in order to preserve data privacy. As a result, any queries that require IPD access—such as identifying specific subpopulations based on age or their respective lab value thresholds—are not supported. For example, identifying participants aged 60–75 who meet a laboratory threshold and are receiving medication X—are not supported. Such queries are possible only when (i) IPD are accessible, or (ii) the metadata expose an appropriate joint (cross-tabulated) summary across age $\times$ laboratory value $\times$ medication for each participant in this group.

CMEO focuses on semantic description and discovery; it does not infer threshold-based clinical classifications unless explicitly specified and agreed upon, nor does it transform IPD. Such functions reside in an interpretive layer implemented as project-specific, auditable rules with explicit provenance to clinical guidelines.

## Related work

The metadata-driven exploration and harmonization across diverse studies remain critical challenges, primarily due to the lack of structured, interoperable frameworks. Current CDMs and ontologies often overlook comprehensive support for essential components such as detailed study design specifications, robust variable-level metadata definitions, explicit statistical profiling, and precise data use permissions. The SDTM standard by CDISC [13] provides structured formats for clinical trial data submission, while ODM [14] defines a metadata and data interchange standard that facilitates study design representation, execution, and archiving. However, both standards primarily address interventional studies and are less effective for observational or epidemiological research, where metadata requirements differ significantly.

PCORnet [15] and OMOP CDM [4, 5] are designed primarily for IPD harmonization across institutions. PCORnet standardizes patient-level content—demographics, encounters, diagnoses, procedures, medications—to support comparative effectiveness research. OMOP likewise normalizes clinical observations, conditions, and interventions for large-scale analytics and multi-site studies. However, because both models target patient-level data integration, they provide only basic metadata and offer limited support for variable-level metadata.

Croissant ML is a schema.org–aligned JSON-LD format that standardizes dataset descriptions for machine-learning workflows across repositories and tools [55]. It has four layers for metadata description including dataset metadata layer, resource layer, structure layer, and light Semantic layers. It provides dataset card for any dataset—files/resources and tables/fields. It does not provide representation for specific metadata including study design, protocol, selection criteria or participants information. In contrast, CMEO is narrower and study-centric.

Some ontology-based frameworks such as the OBI [16] and STATO [17] support structured metadata representation at conceptual levels. STATO emphasizes statistical methods representation and analysis concepts crucial for clinical research metadata. Yet, they have not been explored to addresses unified metadata management or provides explicit semantic linkages necessary for comprehensive metadata management. The DUO ontology [21] specifically addresses data permissions and access restrictions in biomedical research, providing standardized terms and relationships for clearly communicating data reuse conditions. Although the DUO ontology provides a valuable framework for data governance, it has seen limited adoption within the metadata-driven exploration of clinical research catalogs.

Several other ontology-based efforts have been proposed to improve metadata representation and management. The Clinical MetaData Ontology (CMO) [56] classifies clinical data elements semantically but lacks comprehensive structural relationships and detailed descriptive information about study design. The Clinical Data Integration Model (CDIM) [57] facilitates structural integration across primary care datasets but provides limited descriptive and governance metadata support. MedRed Ontology [58] emphasizes provenance metadata, covering structural and partially descriptive metadata with no support for data analysis related representation. OMeta, tailored specifically to omics studies, effectively manages descriptive metadata related to biological samples and experimental procedures but lacks comprehensive structural and governance integration [59]. MDRBridge [60] utilizes ISO 11179-compliant templates primarily for practical structural metadata capture but lacks ontology-based semantic consistency, descriptive granularity, and governance metadata management.

The Ontology of Clinical Research (OCRe) [61] is an OWL-2 ontology for human studies that models study-protocol structure, including design types, interventions, and outcomes. It uses the Eligibility Rule Grammar and Ontology (ERGO) [62] to represent eligibility criteria. Similarly, the Clinical Trial Ontology (CTO) [63] provides a BFO-compliant, PICO-centred model of clinical-trial protocols—covering registry identifiers, roles, status and dates, and outcome specifications—with demonstrated uses in chemical-knowledge linking and literature mining. Neither OCRe nor CTO includes detailed representations of dataset metadata or governance information for studies.

OntoStudyEdit [64] introduces detailed descriptive metadata structures for clinical and epidemiological metadata but does not adequately address structural relationships or governance aspects. PACIFIC ontology integrates heterogeneous cardiology data effectively structurally and descriptively but provides limited explicit representation of governance metadata [65]. The CARE semantic model [66], developed for representing data elements in rare disease registries, offers descriptive metadata representation but lacks support for statistical characterization and governance-related metadata. The Ontology of Precision Medicine and Investigation (OPMI) [67] is designed to represent clinical case report forms (CRFs) within the context of precision medicine. Its metadata coverage includes partial support for structural metadata, such as the source of electronic health records (EHRs) and record availability. For descriptive metadata, OPMI categorizes CRF-derived data elements into various types, including quality and measurement, conditions, and medical interventions. However, it does not support statistical metadata related to data elements, nor does it include governance metadata concerning access protocols or study availability.

To illustrate the differences among above mentioned metadata models and our approach, Table 1^1^ presents a structured comparison based on the following criteria:

- **Structural Metadata**: It includes study title, study design (clinical trial, observational study etc.), study protocol which has part selection criteria, primary and secondary outcomes, number of participants etc.
- **Descriptive Metadata**: It represents each data element and its attributes—including permissible values, measurement units, temporal references, coding schemes, and categorization—and supports analysis and profiling of variables for research and validation.
- **Governance Metadata**: Integration with access control standards such as the Data Use Ontology (DUO) for defining permissions and data use restrictions.

**Table 11:** Evaluation of existing metadata models by metadata type in clinical studies

Metadata Model	Structural	Descriptive	Governance
**Non-Ontological Models**			
MDRBridge	$\checkmark$	$\times$	$\times$
Croissant ML	*p*	*p*	$\times$
**Ontologies**			
CMO	$\times$	*p*	$\times$
CDIM	$\checkmark$	*p*	$\times$
MedRed Ontology	$\checkmark$	*p*	$\times$
OMeta	*p*	$\checkmark$	$\times$
OCRe	*p*	$\times$	$\times$
CTO	*p*	$\times$	$\times$
OntoStudyEdit	*p*	$\checkmark$	$\times$
OPMI	*p*	*p*	$\times$
PACIFIC Ontology	$\checkmark$	*p*	$\times$
CARE semantic model	$\checkmark$	*p*	$\times$
OBI/STATO	$\checkmark$	*p*	$\times$
Data Use Ontology (DUO)	$\times$	$\times$	$\checkmark$
**CMEO (Our Approach)**	$\checkmark$	$\checkmark$	$\checkmark$

$\checkmark$ = Full coverage, *p* = Partial coverage, $\times$ = No coverage

In summary, existing approaches contribute to organizing clinical data, capturing provenance, and managing access permissions. However, they are often narrow in scope and lack integration across structural, descriptive, and governance metadata. Crucially, they provide limited support for variable comparison and study compatibility. In such cases, metadata becomes the primary source of insight. There is a clear need for a unified, FAIR-aligned framework that supports harmonization through structured, interoperable, and queryable metadata.

## Conclusion

CMEO addresses persistent challenges in the exploration and harmonization of clinical study metadata in contexts where individual-participant data (IPD) are not accessible. By integrating structural, descriptive, statistical, and governance information within a semantically rigorous framework, it enables study discovery and cross-study variable comparison from metadata alone. Building on established ontologies, CMEO formalizes study protocol, variable semantics, exploratory analyses, and data-use permissions. Importantly, CMEO is complementary to downstream transformation processes. It yields harmonization specifications that support discovery, comparability assessment, and planning without IPD. When governed access is granted, these specifications provide a rapid, structured understanding of the available data and its comparability. They also guide the transformation process, reducing implementation burden, strengthening provenance, and promoting cross-site reuse.

CMEO contributes to the broader goal of enabling semantically integrated, metadata-driven research across distributed clinical datasets. As part of ongoing work, CMEO will be used to standardize metadata across all studies within the iCARE4CVD consortium. This use case will inform iterative refinements of the ontology and evaluate its applicability in real-world harmonization scenarios. Future work will (i) operationalize the harmonization-assessment workflow, (ii) design and evaluate a complementary approach to handle context-dependent quantitative correspondences, and (iii) conduct harmonization assessment across multiple studies.

In prospective applications, CMEO can serve as a semantic foundation for generating ontology-based harmonization specifications across studies and for enabling federated learning workflows through metadata-level alignment of study variables. Furthermore, when integrated with large language models, it has the potential to support the automated identification of study-relevant features based on specific research questions and to facilitate their retrieval across heterogeneous datasets—thereby advancing scalable, privacy-preserving cohort construction.

## Electronic supplementary material

Below is the link to the electronic supplementary material.

Supplementary Material 1

## Data Availability

This manuscript does not report data generation or analysis. All metadata used in this study are available within the manuscript and GitHub mentioned.

## Footnotes

1 https://explorer.icare4cvd.eu

## Code availability

Code used to generate the named graphs is archived at 10.5281/zenodo.17455082. The latest development version is at cmeo repository.
