# Supplementary material for: CMEO: a metadata-centric ontology for clinical studies exploration and harmonization assessment
Source: BMC Med Inform Decis Mak. 2025 Dec 6;26:8. doi: 10.1186/s12911-025-03272-5 (PMC12798102; doi:10.1186/s12911-025-03272-5)
Supplement: Supplementary file 1 — Supplementary Material 1 [file 12911_2025_3272_MOESM1_ESM.pdf]

1 Supplementary Material

```
PREFIX obi:  <http://purl.obolibrary.org/obo/obi.owl/>
PREFIX dc:   <http://purl.org/dc/elements/1.1/>
PREFIX bfo:  <http://purl.obolibrary.org/obo/bfo.owl/>
PREFIX cmeo: <https://w3id.org/CMEO/>
PREFIX rdfs: <http://www.w3.org/2000/01/rdf-schema#>
PREFIX ro: <http://purl.obolibrary.org/obo/ro.owl/>
PREFIX rdf:  <http://www.w3.org/1999/02/22-rdf-syntax-ns#>
PREFIX iao:   <http://purl.obolibrary.org/obo/iao.owl/>
PREFIX stato: <http://purl.obolibrary.org/obo/stato.owl/>
PREFIX obcs: <http://purl.obolibrary.org/obo/obcs.owl>
PREFIX sio: <http://semanticscience.org/ontology/sio.owl/>
PREFIX xsd: <http://www.w3.org/2001/XMLSchema#>
PREFIX duo: <http://purl.obolibrary.org/obo/duo.owl/>

SELECT
    ?study_name
(GROUP_CONCAT(DISTINCT COALESCE(?part_uri); separator=";") AS ?
    all_parts_uri)
(GROUP_CONCAT(DISTINCT COALESCE(?part_value); separator=";") AS ?
    all_parts_value)

WHERE {
  GRAPH <https://w3id.org/CMEO/graph/studies_metadata> {
      ?study  a cmeo:randomized_controlled_trial;
                      ro:has_part   ?protocol;
              dc:identifier ?study_name.
      ?protocol a obi:protocol;
              (ro:has_part)+ ?part_uri .
      ?part_uri cmeo:has_value ?part_value.



  }
}
GROUP BY ?study_name
```

Listing 1 CQ1: What are the protocol specifications of the randomized controlled trials?

```
SELECT DISTINCT ?study_name ?description
WHERE {
    GRAPH <https://w3id.org/CMEO/graph/studies_metadata> {
        ?study_design dc:identifier ?study_name;
            iao:is_about ?descriptor;
            ro:has_part ?prot .
        ?descriptor rdfs:label ?description.
        ?prot a obi:protocol;
```

```
        ro:has_part ?obj_spec.
      ?obj_spec a obi:objective_specification ;
          cmeo:has_value ?obj_val.
      ?prot ro:has_part ?ec.
      ?ec a obi:eligibility_criterion;
          ro:has_part ?inc.
      ?inc a obi:inclusion_criterion;
          ro:has_part ?spec_inc.
      ?spec_inc  cmeo:has_value ?inc_value.

    FILTER(contains  (?obj_val,"heart failure") || contains  (?
       inc_value,"heart failure"))

  }
}
```

Listing 2 CQ2: Which studies investigated heart failure condition?

```
SELECT ?study_name (GROUP_CONCAT(DISTINCT LCASE(STR(?
    quality_value)); separator="; ") AS ?qualities)
(GROUP_CONCAT(DISTINCT LCASE(STR(?quality_type)); separator="; ")
     AS ?quality_type)
WHERE {
GRAPH <https://w3id.org/CMEO/graph/studies_metadata> {
        ?study_design dc:identifier ?study_name ;
           ro:has_part ?prot .
       ?prot a obi:protocol ;
           ro:has_part ?ec .
       ?ec a obi:eligibility_criterion ;
           ro:is_concretized_by ?hse_uri .
       ?hse_uri ro:has_output ?population .
       ?population a obi:population ;
           ro:has_characteristic ?quality .
       OPTIONAL { ?quality rdfs:label ?characteristic_value }
       OPTIONAL { ?quality a  ?characteristic_type }
}
}

GROUP BY ?study_name
LIMIT 3
```

Listing 3 CQ3: What are the population characteristics in different studies

---

```
SELECT DISTINCT ?study_name
(GROUP_CONCAT(DISTINCT CONCAT(LCASE(STR(?diagnosis_value)), " [",
     STR(?min_val), "-", STR(?max_value), "]"); SEPARATOR="; ") AS
     ?population)
```

```
(GROUP_CONCAT(DISTINCT LCASE(STR(?intervention_value)); separator
    ="; ") AS ?interventions)
(GROUP_CONCAT(DISTINCT LCASE(STR(?comparator_value)); separator="
    ; ") AS ?comparators)
(GROUP_CONCAT(DISTINCT LCASE(STR(?outcome_value)); separator="; "
    ) AS ?outcomes)

WHERE {
 GRAPH <https://w3id.org/CMEO/graph/studies_metadata>

 {

    ?study_design dc:identifier ?study_name;
          ro:has_part ?prot .
    ?prot a obi:protocol;
        ro:has_part ?eligibility_criterion .
    ?eligibility_criterion a obi:eligibility_criterion ;
        ro:has_part ?inclusion_criteria .
    ?inclusion_criteria a obi:inclusion_criterion ;
        ro:has_part ?inc_part.
    #DIAGNOSTIC INCLUSION
    OPTIONAL{

     ?inc_part a obi:health_status_inclusion_criterion;
           cmeo:has_value ?diagnosis_value .
     FILTER(contains  (?diagnosis_value, "nyha class ii"))
      }
      #AGE INCLUSION
      OPTIONAL{
         ?inc_part a obi:age_group_inclusion_criterion;
            ro:has_part ?min_val_specification, ?
               max_value_specification.
          ?min_val_specification a obi:
              minimum_age_value_specification;
               cmeo:has_value ?min_val.
          ?max_value_specification a obi:
              maximum_age_value_specification;
               cmeo:has_value  ?max_value.
          FILTER(xsd:integar(?min_val) >= 60 && xsd:integar(?
              max_value) <= 85)
      }
     # Intervention
        OPTIONAL{
        ?prot ro:has_part ?intervention .
        ?intervention a cmeo:intervention_specification; cmeo:
            has_value ?intervention_value .
        FILTER(contains  (?intervention_value,"intensified"))
     }
     OPTIONAL{
        # Comparator
```

```
        ?prot ro:has_part ?comparator .
        ?comparator a cmeo:comparator_specification ;cmeo:
            has_value ?comparator_value .
        FILTER(contains  (?comparator_value,"standard guided"))
        # Outcomes
     }
      OPTIONAL{
        ?prot ro:has_part ?outcome .
        ?outcome a cmeo:outcome_specification ;
                ro:has_part ?suboutcome_uri.
        ?suboutcome_uri  cmeo:has_value ?outcome_value .
        FILTER(contains  (?outcome_value,"survival"))
    }
}
GROUP BY ?study_name
```

Listing 4 CQ4: Which studies match the PICO criteria ?

```
SELECT DISTINCT ?variable ?missing_value
WHERE {
    GRAPH <.../time-chf>  {
        ?dataElement a cmeo:data_element;
            dc:identifier ?variable ;
         obi:has_value_specification ?missingSpec .
        ?missingSpec a cmeo:missing_value_specification;
            cmeo:has_value ?missing_value .
}
}
LIMIT 3
```

Listing 5 Q5: How are missing values encoded in study A?

```
SELECT ?variable ?fq_value
WHERE {
    GRAPH <https://w3id.org/CMEO/graph/time-chf> {
                ?dataElement a cmeo:data_element ;
             dc:identifier ?variable.

        ?statistical_variable a ?type;
                iao:denotes ?dataElement.
        VALUES ?type { cmeo:binary_class_variable cmeo:
            multi_class_variable }

        ?ds a iao:dataset ;
             iao:is_about ?statistical_variable ;
             obi:is_specified_input_of ?eda_proc .

         ?eda_proc a cmeo:exploratory_data_analysis;
            obi:has_specified_output ?stats .
```

```
        ?stats a stato:statistic ;
            ro:has_part ?part_uri .
      OPTIONAL{
        ?part_uri a obcs:frequency_distribution;
            cmeo:has_value ?fq_value .
    }

  }
}
LIMIT 3
```

Listing 6 CQ6: Which categorical variables does Study A include, and what are their distributions?

```
SELECT
?omop_id ?code_label ?code_value ?val
(GROUP_CONCAT(DISTINCT ?varNameA; SEPARATOR=", ") AS ?source)
(GROUP_CONCAT(DISTINCT ?varNameB; SEPARATOR=", ") AS ?target)
(GROUP_CONCAT(DISTINCT ?visitsA ; SEPARATOR=", ") AS ?
    source_visit)
(GROUP_CONCAT(DISTINCT ?visitsB ; SEPARATOR=", ") AS ?
    target_visit)
WHERE {
{
    # ---------- TIME-CHF (source) ----------
    SELECT
    ?omop_id ?code_label ?code_value ?val
    (GROUP_CONCAT(DISTINCT ?var_nameA; SEPARATOR=", ") AS ?
        varNameA)
    (GROUP_CONCAT(DISTINCT ?pairA   ; SEPARATOR=", ") AS ?visitsA
        )
    ("time-chf" AS ?source)
    WHERE {
    GRAPH <https://w3id.org/CMEO/graph/time-chf>  {
        # 1) Most selective: mapping from data element ->
            standardized code (rdf:_1) -> OMOP
        ?stdProcessA a cmeo:data_standardization ;
                    obi:has_specified_output ?codeSetA ;
                    obi:has_specified_input  ?dataElementA .
        ?codeSetA rdf:_1 ?codeNodeA .
        ?codeNodeA a cmeo:code ;
                cmeo:has_value ?code_value ;
                rdfs:label     ?code_label ;
                iao:denotes    ?omopClassA .
        ?omopClassA a cmeo:omop_id ; cmeo:has_value ?omop_id .

        # 2) Data element identity (single valued)
        ?dataElementA a cmeo:data_element ; dc:identifier ?
            var_nameA .
```

```
        # 3) Optional category value (kept single per DE; if
            multi, choose one deterministically)
        OPTIONAL {
        ?catProcessA a cmeo:categorization_process ;
                    obi:has_specified_input  ?dataElementA ;
                    obi:has_specified_output ?catOutA .
        ?catOutA cmeo:has_value ?val .
        }
        OPTIONAL {
        {
            SELECT ?dataElementA (GROUP_CONCAT(DISTINCT ?
                visitLblA; SEPARATOR="|") AS ?visitStrA)
            WHERE {{
            ?visitDatumA a cmeo:visit_measurement_datum ;
                        iao:is_about ?dataElementA ;
                        obi:is_specified_input_of ?vsProcA .
            ?vsProcA obi:has_specified_output ?visitCodeA .
            ?visitCodeA rdfs:label ?visitLblA .
            }}
            GROUP BY ?dataElementA
        }
        }

        # 5) Make the (var || visits) pair once, not
            multiplicatively
        BIND(COALESCE(?visitStrA, "") AS ?visA)
        BIND(CONCAT(STR(?var_nameA), "||", ?visA) AS ?pairA)
    }
    }
    GROUP BY ?omop_id ?code_label ?code_value ?val
}
UNION
{
    # ---------- GISSI-HF (target) ----------
    SELECT
    ?omop_id ?code_label ?code_value ?val
    (GROUP_CONCAT(DISTINCT ?var_nameB; SEPARATOR=", ") AS ?
        varNameB)
    (GROUP_CONCAT(DISTINCT ?pairB   ; SEPARATOR=", ") AS ?visitsB
        )
    ("gissi-hf" AS ?target)
    WHERE   {
    GRAPH <https://w3id.org/CMEO/graph/gissi-hf> {
        ?stdProcessB a cmeo:data_standardization ;
                    obi:has_specified_output ?codeSetB ;
                    obi:has_specified_input  ?dataElementB .
        ?codeSetB rdf:_1 ?codeNodeB .
        ?codeNodeB a cmeo:code ;
                cmeo:has_value ?code_value ;
```

```
            rdfs:label      ?code_label ;
            iao:denotes     ?omopClassB .
    ?omopClassB a cmeo:omop_id ; cmeo:has_value ?omop_id .

    ?dataElementB a cmeo:data_element ; dc:identifier ?
       var_nameB .

    OPTIONAL {
    ?catProcessB a cmeo:categorization_process ;
                obi:has_specified_input  ?dataElementB ;
                obi:has_specified_output ?catOutB .
    ?catOutB cmeo:has_value ?val .
    }

    OPTIONAL {
    {
        SELECT ?dataElementB (GROUP_CONCAT(DISTINCT ?
           visitLblB; SEPARATOR="|") AS ?visitStrB)
        WHERE {
        ?visitDatumB a cmeo:visit_measurement_datum ;
                    iao:is_about ?dataElementB ;
                    obi:is_specified_input_of ?vsProcB .
        ?vsProcB obi:has_specified_output ?visitCodeB .
        ?visitCodeB rdfs:label ?visitLblB .
        }
        GROUP BY ?dataElementB
    }
    }

    BIND(COALESCE(?visitStrB, "") AS ?visB)
    BIND(CONCAT(STR(?var_nameB), "||", ?visB) AS ?pairB)
  }
  }
  GROUP BY ?omop_id ?code_label ?code_value ?val
}
}
GROUP BY ?omop_id ?code_label ?code_value ?val
ORDER BY ?omop_id
```

Listing 7 CQ7: Which variables are similar between selected studies?

```
SELECT DISTINCT (?g as ?study_name) ?var_name ?device_id
WHERE {
  GRAPH ?g {
    # Start with the data collection process
    ?dacq_proc a obcs:data_collection;
               obi:has_specified_input ?device;      # Find the
                  device used AS INPUT by the process
               obi:has_specified_output ?ds.
```

```
    # Now, qualify the device and get its info
    ?device a cmeo:wearable_device;
            cmeo:has_value ?device_id.

    # Continue the chain from the process output to the variable
       name
    ?ds a iao:dataset;
        iao:is_about ?stat_var.

    ?stat_var iao:denotes ?de.

    ?de a cmeo:data_element;
        rdfs:label ?var_name.

    # (Optional) You can still ensure the device has a sensor if
       needed
    # This is now just a filter, not part of the main traversal
       path
    ?device ro:has_part ?sensor.
  }
}
LIMIT 5
```

Listing 8 CQ8: Which studies have variables measured with wearable devices?

```
SELECT ?study_name
WHERE {
  GRAPH <https://w3id.org/CMEO/graph/studies_metadata> {

    ?assignment a cmeo:data_use_permission_assignment ;
                iao:is_about ?x_study ;
                obi:has_specified_output ?data_policy ;
                obi:has_specified_output ?data_modifier .
    ?x_study a obi:study_design_execution ;
             dc:identifier ?study_name .
    ?data_policy a duo:data_use_permission ;
                 rdfs:label "disease specific research"^^xsd:
                    string ;
                 iao:is_about ?disease_code .
    ?data_modifier a duo:data_use_modifier ;
                   rdfs:label "ethics approval required"^^xsd:
                      string .

    OPTIONAL { ?disease_code rdfs:label "congestive heart failure
       "^^xsd:string }
    OPTIONAL { ?disease_code cmeo:has_value "42343007"^^xsd:
       string }
  }
```

```
}
ORDER BY ?study_name
```

Listing 9 CQ9: What studies permit data access for research on congestive heart failure, subject to ethics-approval?
